# Draft Genome Sequence of the *Mycobacterium tuberculosis* Clinical Isolate C2, Belonging to the Latin American–Mediterranean Family

**DOI:** 10.1128/genomeA.00536-14

**Published:** 2014-06-05

**Authors:** Yu-Chieh Liao, Yih-Yuan Chen, Hsin-Hung Lin, Jia-Ru Chang, Ih-Jen Su, Tsi-Shu Huang, Horng-Yunn Dou

**Affiliations:** a National Institute of Infectious Diseases and Vaccinology, National Health Research Institutes, Zhunan, Miaoli, Taiwan; b Division of Biostatistics and Bioinformatics, Institute of Population Health, National Health Research Institutes, Zhunan, Miaoli, Taiwan; c Department of Microbiology, Kaohsiung Veterans General Hospital, Kaohsiung, Taiwan

## Abstract

Tuberculosis remains a major infectious disease in Taiwan. Here we present the draft genome sequence of the *Mycobacterium tuberculosis* C2 strain, belonging to the Latin American–Mediterranean lineage. The draft genome sequence comprises 4,453,307 bp with a G+C content of 65.6%, revealing 4,390 coding genes and 45 tRNA genes.

## GENOME ANNOUNCEMENT

Tuberculosis is a leading notifiable communicable disease in Taiwan. Based on molecular methods, six distinct clades, Beijing, Haarlem, East-African Indian (EAI), Latin American–Mediterranean (LAM), U, and T lineages, were isolated. Epidemiological surveillance indicated that the Beijing lineage was the most prevalent strain in Taiwan, followed by EAI and Haarlem strains (1, 2). The percentages of isolated LAM strains were around 1% to 7% (2, 3).

Here we report the complete genome sequence of a clinical isolate of *Mycobacterium tuberculosis* strain C2, isolated at Kaohsiung Veterans General Hospital, Taiwan, from human sputum. Sputum microscopy and culture samples from the patient tested positive for tuberculosis. The genomic DNA of isolate C2 was extracted from cultured cells as described previously (4), and was sequenced using a MiSeq platform (Illumina, San Diego, CA, USA). Strain C2 was also analyzed by spoligotyping (337777607760771) and mycobacterial interspersed repetitive unit-variable number tandem repeat (MIRU-VNTR) typing (2241243261532241144132250) and was identified as a member of the LAM family. The LAM lineage was one of six major clades of *M. tuberculosis* strains identified in Taiwan. LAM originating in Europe and America may have first occurred during the Portuguese reign in the 16th century and been passed on to the natives of Taiwan.

This study was approved by the Human Ethics Committee of the National Health Research Institutes, Taiwan (code EC1010804-E). Because of the retrospective nature, routine collection of clinical data in daily practice, and dislinkage of personal information, the requirement to obtain informed consent was waived by our institutional review board.

A total of 8,259,434 paired-end reads of 251 bp in length, with an average insert size of 289 bp, were produced. The sequencing reads were trimmed and discarded by limiting the quality score to 0.05 and permitting at most two ambiguous nucleotides in the minimum length of 50 bp. Five different genome assemblies generated separately by using Abyss v1.3.4 (5), Edena v3.130110 (6), SPAdes v2.5.0 (7), and Velvet v1.2.09 (8), were subsequently integrated into an assembly by using CISA (9). The assembly resulted in 85 contigs (>200 bp) at *N*_50_ value 168,084 bp and a total assembly of 4,453,307 bp, with a G+C content of 65.6%. The draft genome was annotated using the RAST server (10) resulting in a total of 4,390 coding genes and 45 tRNA genes.

### Nucleotide sequence accession numbers.

This whole-genome shotgun project has been deposited at DDBJ/EMBL/GenBank under accession no. JHAD00000000. The version described in this paper is the first version, JHAD01000000.
